# Humor at work that works: A multi-level examination of when and why leader humor promotes employee creativity

**DOI:** 10.3389/fpsyg.2022.903281

**Published:** 2022-08-01

**Authors:** Yajun Zhang, Changqin Yin, Muhammad Naseer Akhtar, Yongqi Wang

**Affiliations:** ^1^School of Business Administration, Guizhou University of Finance and Economics, Guiyang, China; ^2^School of Management, Wuhan Polytechnic University, Wuhan, China; ^3^Royal Docks School of Business and Law, The University of East London, London, United Kingdom; ^4^International College, Guangdong University of Foreign Studies, Guangzhou, China

**Keywords:** leader humor, creativity, creative self-efficacy, traditionality, cultural representation theory

## Abstract

Although the role of leadership in fostering employee creativity has been extensively studied, it is still unclear whether and how leader humor affects employee creativity. Drawing upon cultural representation theory (CRT), we examined creative self-efficacy as a mediator and traditionality as a situational factor in the relationship between leader humor and employee creativity by analyzing a sample of 306 employees and 88 leaders (paired data) collected through survey questionnaire from firms based in Hubei Province, China, covering the industries of automobile, IT, and medicine. Following the multi-level examination, leader humor was positively related to employee creativity, and creative self-efficacy was found to mediate the impact of leader humor on employee creativity. Furthermore, traditionality moderated the effect of leader humor on creative self-efficacy, as well as the indirect effect of leader humor on employee creativity through creative self-efficacy. This study provides a social psychological explanation for the association between humor and employee creativity, deepens the current understanding of the influence process of leader humor. Finally, the theoretical and practical implications of the study are discussed at the end alongside limitations and recommendations for future research.

## Introduction

Workplace creativity is the generation of original and constructive ideas or solutions (Shalley, 1991; Oldham and Cummings, 1996; Tang et al., 2017; Akkan and Guzman, 2022). Similarly, employee creativity is an important driving force for fostering organizational innovation, maintaining competitive advantage, and achieving success (Liu et al., 2016). Given the importance of employee creativity in organizations, researchers have conducted extensive research on the factors that influence employee creativity. Leaders as an important object with which employees come into contact in the workplace, have a significant impact on employees’ creativity. As a result, both academia and practice have paid close attention to the relationship between leader behavior and employee creativity (Amabile et al., 2004). The literature on several facets of leadership showed that transformational leadership (Gong et al., 2009; Shafi et al., 2020), innovation leadership (Tung and Yu, 2016; Kremer et al., 2019), empowering leadership (Zhang and Zhou, 2014), moral leadership (Gu et al., 2015; Feng et al., 2018), servant leadership (Jaiswal and Dhar, 2017), and authentic leadership (Xu et al., 2017; Guo et al., 2018) are important factors in promoting employee creativity.

Despite the fact that leader humor is thought to be an effective tool for cultivating a creative environment (Amjed and Tirmzi, 2016; Peng et al., 2020; Yang et al., 2021; Fu et al., 2022), the influence of leader humor on employee creativity is rarely investigated. The few scholars who have studied the relationship between leader humor and employee creativity put leaders and employees at the same level of research, i.e., individual level, and the theoretical perspective of the research is limited to the relatively single perspective, i.e., social exchange theory. For example, Li et al. (2019) found that leader humor can promote employee creativity both directly and indirectly (*via* psychological capital), but there is no theoretical support. Lang and Lee (2010) investigated the direct impact of leader humor on employee creativity using social exchange theory examined both leader humor and employee creativity as same level constructs, i.e., individual level analysis. However, Cooper et al. (2018) studied leader humor by integrating social exchange theory, conservation of resources theory, and broaden-and-build theory, but their research focused on employee citizenship behavior rather than creativity.

These studies contributed to the advancement of research on the relationship between leader humor and employee creativity. However, because leaders and employees are at different levels of the organization, thus, a cross-level investigation can provide a more accurate understanding of the relationship between leader humor and employee creativity. At the same time, the relationship between leader humor and employee creativity needs to be explained using theories that are appropriate for the various research scenarios and samples. For instance, social exchange theory does not account for the large variations in how different cultural value orientations influence the behavior of employees, even those who work in the same country and society. Therefore, the cross-level analysis of how leader humor promotes employee creativity is the first research gap that this study focuses on through the lens of cultural self-representation theory.

Cultural self-representation theory holds that the work environment, such as management techniques can affect individual behavior by influencing an individual’s self-concept (Erez and Earley, 1993). As an important part of individual self-concept, creative self-efficacy is the employees’ subjective evaluation of their ability to be creative in a specific duty or work (Tierney and Farmer, 2002), which is important for the actual delivery of employee creativity. Meanwhile, creative self-efficacy is influenced by personality traits and environmental factors (Mathisen, 2011). In an organization, a leader’s behavior is a crucial environmental factor and has an important impact on employees’ self-concept and behavior. Therefore, we can infer that the association between leader humor and employee creativity is mediated by creative self-efficacy. Although, existing literature showed that relational energy (Yang et al., 2021), psychological capital (Li et al., 2019), task resources, and affective commitment (Hu and Luo, 2020) are important mediating variables in the process of workplace humor promoting employee creativity. However, they have neglected the significant role of individual self-concept between leader behavior and employee creativity. Thus, the second research gap that this study focuses on is the neglect of the mediating role of employee creative self-efficacy between leader humor and employee creativity.

Furthermore, academics call for organizational management research to be carried out in a specific context. The cultural representation theory contends that an individual’s cultural beliefs determine how the workplace affects that person’s self-concept and behavior (Newman and Nollen, 1996). Hence, it is most likely that cultural values will play a significant situational role in how leader humor affects employee creativity. Existing research has produced some conclusions and found that perspective-taking (Hu and Luo, 2020), employees’ sensitivity to favorable interpersonal treatment (Peng et al., 2020), supervisor-subordinate dyadic tenure, and work autonomy (Li et al., 2019) are significant situational factors affecting the relationship between leader humor and employee creativity, but it has neglected the influence of individual cultural values in the relationship. Individual traditionality is an important indicator to measure the degree of the influence of a specific traditional cultural value on an individual (Farh et al., 1997). in general, a person is more likely to submit to authority, observe the law, practice conservatism, and put up with the actions of leaders the more traditional they are. As a result, high-individual traditional subordinates adhere more to the hierarchical relationship between leader and subordinate than low-individual traditional subordinates (Farh et al., 2007). Hence, we argue that individual traditionality may have a moderating impact on creativity and self-efficacy. The third research gap that this study focuses on is the neglect of the individual’s traditionality in previous studies. In order to better understand how leader humor affects creative self-efficacy and how it affects employee creativity *via* creative self-efficacy, we tested the modest role of individual traditionality.

**Figure 1:**
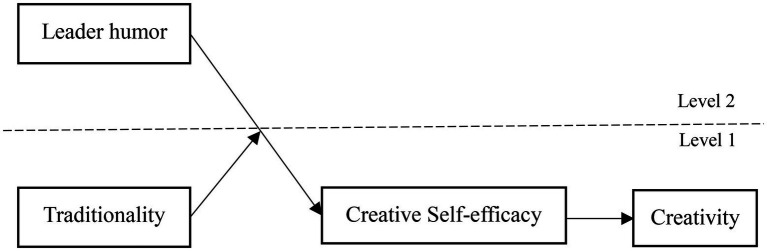
Theoretical model.

In conclusion, our study is aimed at examining when and why leader humor affects employee creativity. To test the theoretical conjectures and to fill the research gaps identified in this study, we developed a research model (please see Figure 1) based on the cultural self-representation theory and proposed hypotheses. To test the hypothesis, the researchers then performed a multi-wave survey questionnaire and collected paired data from leaders and subordinates. Our research makes the following key contributions. First, this study broadens the application and scope of cultural self-representation theory and advances our knowledge of the social psychological process through which leader humor affects workers’ creativity. Second, this study advances the study of the mediating mechanism by which leader humor influences employee creativity by introducing creative self-efficacy as a mediator between leader humor and employee creativity and deepens our understanding on such mediating mechanism. Finally, this study investigates the moderating role of traditionality, advancing boundary-condition research on the impact of leader humor on employee creativity.

In the remainder of the article, the second part presents the theoretical background and the research hypotheses, the third part introduces the research methods, and the fourth part demonstrates the results and discusses the findings of the study, the fifth part introduces the theoretical and practical implications, alongside limitations of this study as well as a conclusion in the end.

## Theoretical background and research hypotheses

### Cultural self-representation theory

Cultural self-representation theory (CRT) proposes a culture-based model of work motivation to evaluate the potential impact of management techniques or practices on employee work motivation in different cultures (Erez and Earley, 1993). As per CRT, individuals independently process self-relevant information in their environment in accordance with the extent to which it contributes to their own values and interests. This means that an individual’s work environment can have an impact on their behavior and self-concept. At the same time, cultural values play an important role in the processing of self-relevant information, determining how employees conceptualize management practices and incorporate them into their self-concept. Therefore, the cultural self-representation theory serves as the theoretical basis of this study, which provides the basic assumptions for the study: (1) The work environment, such as management techniques will affect the individual’s self-concept and behavior; (2) The degree of influence of work environment on individual self-concept and behavior depends on cultural value.

### Leader humor and employee creativity

Employee creativity refers to the ability of employees to generate new ideas, discover and create new things (Lee and Kim, 2021). Employee creativity largely depends on the cognitive characteristics of the individual, as it is often described as an intra-individual cognitive process that breaks habitual mental stereotypes. However, creativity is not an innate and hard-to-change personal trait, but can be nurtured and developed within specific organizational and cultural contexts (Amabile et al., 2004). This is in line with the view of Erez and Earley’s (1993) cultural self-representation theory that the organization’s environment and culture can affect employees’ job performance, such as creativity.

Cooper et al. (2018) believe that leader humor refers to the use of humor by leaders to subordinates. Based on the definition of humor by Cooper et al. (2018), this study interprets leader humor from the perspective of cultural self-representation theory. Cultural self-representation theory emphasizes that the work environment, such as management practices can have a significant impact on personal behavior by affecting the individual’s self-concept. So, we can infer that as a unique interpersonal interaction management style, leader humor has an important influence on employees’ self-concept and behavior. Accordingly, we argue that leader humor is an intentional form of interpersonal humor that leaders use to strengthen their relationships with their subordinates, which might have an impact on the subordinates’ behaviors and self-concepts.

Humor is a powerful form of interpersonal interaction through which leaders can build good relationships with employees, help employees relieve stress, and induce positive emotions in employees to help them address work-related cognitive and emotional challenges (Cooper et al., 2018; Plessen et al., 2020). According to the cultural self-representation theory, leader humor as an important organizational environmental factor affecting employee behavior is expected to have a significant impact on employee creativity. Leader humor can promote and enhance employee creativity through three main functions including relationship building, stress relief, and the induction of positive emotions (Cooper et al., 2018; Lau et al., 2022). First, leaders’ humorous language and behavior can create a favorable environment for employee creativity by building good relationships with subordinates. Employee creative ideas are risky, leader humor helps build an atmosphere that encourages employee innovation (Lang and Lee, 2010; Jiang et al., 2019) and motivates employees to take risks in solving problems by creating, sharing, and executing creative ideas (Janssen et al., 2004; Volery and Tarabashkina, 2021). Leader humor reduces employees’ concerns about creativity risks (Carmeli et al., 2010) and diverts employees’ attention from risks to opportunities (Pundt, 2015). At the same time, humor can continue the idea generation process (Holmes, 2007), so leader humor plays an important role in fostering employee creativity (Slatten et al., 2011). Second, leader humor can promote creativity by relieving employee stress. People under stress tend to repeat normalized behaviors while ignoring or avoiding novel approaches (Hon et al., 2013). Stress makes people less active at work and less enthusiastic when looking for alternatives or trying creative solutions (Ford, 1996). In short, stress hinders creativity. However, humor is an antidote to stress, or at least buffers its harmful effects. It not only increases the positive effects but also neutralizes the negative emotions produced by stressors while distracting and reducing the negative effects of stress (Martin and Lefcourt, 1983). Therefore, humor can boost creativity by reducing employee stress (Fredrickson, 2003; Lang and Lee, 2010). Finally, leader humor can also boost employee creativity by inducing positive emotions in employees. Positive emotions can broaden people’s thinking patterns and make them more creative (Fredrickson, 2003; Madrid and Patterson, 2016). Leaders’ humorous language and behavior can bring joy to their team members (Cooper et al., 2018) and trigger highly activated positive emotions (Russell, 2003), thereby enhancing employee creativity. Thus, we suggest the following hypothesis:

*H1*: Leader humor exerts a positive effect on employee creativity.

### Creative self-efficacy as a mediator

Creativity self-efficacy is considered to be the degree of confidence that individuals have in their ability to complete innovative works, which can provide them the inner drive they need to engage in creative activities (Zhang and Zhou, 2014; Shaw et al., 2021). Individuals high in creative self-efficacy have more confidence in their creative thinking, ability, and believe that they can successfully navigate challenges that arise throughout the creative process (Wang et al., 2014). Conversely, individuals with a low level of creative self-efficacy are more conservative, and have lack of confidence in their creative ability to achieve goals, and are reluctant to take the initiative to try or implement new ideas (Gong et al., 2009). Therefore, it is anticipated that having a strong sense of creative self-efficacy will greatly enhance personal creativity.

The cultural self-representation theory suggests that the work environment can have a significant impact on personal behavior by affecting the individual’s self-concept (Erez and Earley, 1993). So, creative self-efficacy as an important content of self-concept is influenced by the organizational environment such as leader humor (Mathisen, 2011). Specifically, leader humor can promote the exchange of socio-emotional resources with subordinates, generate an ideal relationship with employees featuring mutual trust, respect, and affection, thus, making employees more confident in creative problem-solving (Theeboom et al., 2014). Second, the higher the frequency of humorous communication between leaders and subordinates, the more likely subordinates are to perceive the leader’s support and friendliness. It is conducive to the expression of employees’ wishes, and the shortening of the social distance between leaders and employees. Consequently, employees can enjoy the supportive environment required for creative work, and boost their confidence in completing creative and challenging tasks, thereby stimulating employee creativity (Robert et al., 2016). At the same time, the notion that humorous leaders can inspire positive emotions implies that the exposure to leader humor can encourage subordinates to diversify their thought-action processes and enrich their cognitive, social, and psychological resources (Fredrickson, 2003). Employees that are given additional resources can overcome challenges that arise during the innovation process and have more possibilities to develop their creative abilities. Finally, according to Yuan and Zhou (2008), employees improve their perspectives of creativity in the workplace through imitating others. The workplace culture’s cues for leader behavior have a significant impact on how employees build their own opinions of themselves (Coelho et al., 2011). Humorous leaders can boost their followers’ self-esteem and give them the confidence to complete novel and difficult tasks, which ultimately fosters their creativity. Therefore, we propose the following hypothesis:

*H2*: Employee creative self-efficacy mediates the impact of leader humor on employee creativity.

### Traditionality as a moderator

People’s cultural psychology is generally based on the local culture, and the state is the agent of socio-psychological orientation, leading many researchers to assume that the people of a certain country/region have similar ways of thinking and behavior (Gjerde and Onishi, 2000). Every country has its own traditional cultural values. Traditionality is one of the important indicators to measure an individual’s recognition of traditional values. Highly traditional employees generally have the following five characteristics: obeying authority, filial piety and respecting ancestors, keeping one’s footing, self-preservation, and male superiority (Farh et al., 1997; Xie et al., 2008). In previous studies, traditionality was identified as an important moderator of supervisor-employee relationships, employee self-concept, and organizational behavior relationships (Farh et al., 2007; Wang and Zhang, 2020). Integrating the theoretical perspective of cultural self-representation theory and previous findings, this research suggests that traditionality may moderate the process of leader humor enhancing employees’ creative self-efficacy.

Compared to low-traditionality individuals, individuals high in traditionality tend not to alter their attitudes and behavioral responses according to leaders’ behavior towards’ them. Instead, their attitudes and behaviors are more conditioned by their self-perceived need to meet the expectations and responsibilities of their given social roles (Farh et al., 2007). They are more likely inclined to follow social norms and accept the status quo, e.g., are more confined by role constraints and situational influences. As a result, more traditional employees are less susceptible to leader humor, and stick to conventions and act according to their role in the organization, thereby reducing self-concept and the potential for creative self-efficacy. Second, the core of traditionality is to obey authority (Farh et al., 2007). Employees with high traditionality are reluctant to challenge the power structure of the organization and are keen to maintain hierarchical relationships. They have difficulty in finding the cognitive overlap between themselves and the ambiguous hierarchy of humorous leadership behaviors and are therefore less susceptible to leader humor (Humberd and Rouse, 2016). Hence, compared with individuals with high traditionality, low-traditionality individuals are subjected to a stronger effect of leader humor on subordinates’ thinking, ability, and confidence stimulation. Finally, employees with deep traditional values are more likely to believe in fatalism and have a lower desire for work autonomy (Yang and Cheng, 2009). So, they are less positive about the interpersonal and emotional resources that leader humor can bring. Since they adhere to conventions, self-restraint, and compliance with employer-imposed role norms, employees with high conventionality have little incentive for high levels of expected contribution at work (Xie et al., 2008). Less traditional employees are more willing to express themselves and pursue independence, so they are reluctant to stick to the rules, actively demonstrate their abilities, and can obtain emotional and interpersonal resources from leader humor that may help develop self-creative self-efficacy (Humberd and Rouse, 2016). Therefore, we propose the following:

*H3*: Traditionality negatively moderates the link between leader humor and employee creative self-efficacy.

Similarly, traditionality will also weaken the mediating role of creative self-efficacy in the process of leader humor affecting employee creativity, as it may limit the cues for employee self-concept expression. Specifically, employees who are deeply influenced by traditional values are more likely to confront leaders based on a perception of role responsibilities and obligations in the organization, rather than a perception of the incentive/contribution balance (Farh et al., 2007). Therefore, leader humor may not enhance creative self-efficacy for those employees who are deeply influenced by traditional values because they are more self-contained and tend to accept the status quo. In contrast, low-traditionality workers respond more to leader humor (Farh et al., 2007). High-traditionality employees are less sensitive to leader humor, thus, weakening the stimulating effect of leader humor on their self-efficacy. Lower creative self-efficacy may make employees more conservative, which is ultimately detrimental to creativity. When the traditional level of employees is low, the effect of leader humor in promoting creativity self-efficacy will be stronger. The improvement of creative self-efficacy increases employees’ confidence in their creativity and workability and enhances employees’ creativity, which ultimately enhances the effect of leader humor on the creativity of subordinates. The above discussion leads to the following hypothesis:

*H4*: The indirect effect of leader humor on employee creativity through employee creative self-efficacy is moderated by traditionality, that is, the lower the traditionality, the stronger the indirect effect, and vice versa.

## Materials and methods

### Participants and procedure

This study examines employees (individuals/subordinates) and their immediate supervisors (leaders) of several enterprises (including automobile, IT and medicine) based in Hubei Province, China. Prior to the examination, the researchers and HR managers randomly selected respondents to participate in the survey (Liu et al., 2020). The individuals provided anonymous responses, and the completed questionnaires were coded to guarantee that the same individual’s information was gathered for the subsequent survey. During the data collection process, the researcher was present to distribute and retrieve the questionnaires and informed the subjects that the survey data will only be used for academic discussion and is completely confidential. Each participant who completed the questionnaire was rewarded with the incentive of 50 yuan each.

To reduce the common method bias, we collected data from two sources—employees and their immediate leaders—at three points in time with the interval of 2 weeks. Four hundred employees in 109 teams were randomly selected to participate in the survey questionnaire with the help of aforementioned HR managers. At the first point in time (T1), we required participants to fill out scales on demographic variables, leader humor, and traditionality, 378 questionnaires were returned. At the second point in time (T2), we asked employees who completed the survey in T1 to respond on their creative self-efficacy and positive emotion, 356 questionnaires were returned; At the third point in time (T3), the researchers asked the leaders (immediate/direct supervisors) of employees who completed T2 to rate their employees’ creativity, at this stage 96 questionnaires of team leaders were returned. After excluding the questionnaires that were filled in randomly, with missing data, and impossible to match, a total of 88 team leaders and 306 employees matched samples were obtained. Among them were 156 male employees (50.98%), 116 employees between the ages of 26 and 35 (37.91%), 170 employees with a bachelor’s degree (55.55%), and 116 employees work tenure between 2 and 5 years (37.91%). The demographic variables of this study are shown in Table 1.

**Table 1:** Sample characteristics.

Category	Characteristics	*N*	*%*
Gender	Male	156	50.98
	Female	150	49.02
Age	*≤*25	81	26.47
	26–35	116	37.91
	36–45	55	17.97
	*≥*46	54	17.65
Education level	High school	34	11.11
	Junior college	64	20.92
	Bachelor degree	170	55.55
	Graduate degree	38	12.42
Work tenure	*≤*1	50	16.34
	2–5	116	37.91
	6–10	106	34.64
	*≥*11	34	11.11

N (Level 2) = 88; N (Level 1) = 306.

### Measures

Leader humor: Leader humor is assessed by the 3-item scale developed by Cooper et al. (2018). The sample item includes, e.g., “My leader jokes around with me” (Cronbach *α* = 0.95). And the aggregation indicators ICC (1), ICC (2), and mean Rwg values are 0.59, 0.83, and 0.95, respectively.

#### Creative self-efficacy

We used 3-item scale of Tierney and Farmer (2002) to measure creative self-efficacy, the sample item was, “I have confidence in my ability to solve problems creatively” (Cronbach *α* = 0.90).

#### Traditionality

We measured traditionality with 5-item scale devised by Farh et al. (1997), the sample item was, “Following the instructions of a senior person is the best way to avoid mistakes” (Cronbach *α* = 0.96).

#### Employee creativity

We adapted 4-item scale to measure employee creativity developed by Farmer et al. (2003), the sample item was, “This employee tries new ideas or methods first” (Cronbach *α* = 0.88).

#### Control variables

We have used several control variables in our study. As per the findings of Cooper et al. (2018) that employees’ positive emotions influence creativity. Therefore, this study controlled for employees’ positive emotions and measured them with reference to the 4-item scale of Motro et al. (2021). An example item was “Joyful” (Cronbach *α* = 0.86). At the same time, studies have shown that an individual’s gender, age, education level, and job tenure can affect creativity (Cooper et al., 2018). Therefore, this study also controlled for individuals’ gender, age, education level, and work tenure. All latent variables were marked on a Likert type 5-point scale (1 means strongly disagree; 5 means strongly agree).

### Analysis strategy

In this study, we take employee’s creative self-efficacy, employee creativity, and employee personal tradition (traditionality) as level 1, whereas leader humor as level 2. At the same time, the statistical results showed that creative self-efficacy and employee creativity have obvious between-group differences. Therefore, a multi-level linear model (HLM) was used to analyze the data. Specifically, we first conducted confirmatory factor analysis (CFA) in AMOS v.22 to test the model fitness and discriminant validity, then utilized SPSS v.23 for reliability analysis and descriptive statistics. Second, hypotheses were tested by utilizing multi-level data in HLM v.7. Finally, we carried out moderated mediation analyses in R by following the parametric bootstrap method.

## Results

### Measurement model

To test the discriminant validity of the variables, we carried out confirmatory factor analysis (CFA). The factors with the highest correlation coefficients are combined to build competitive models to compare with benchmark model/baseline model (please see Table 2 for CFA results). Compared with other models, the five-factor model (leader humor, creative self-efficacy, traditionality, positive emotions, and employee creativity) showed the best fit and demonstrated the good discriminant validity.

**Table 2:** Results of confirmatory factor analysis.

**Model**	***χ*** ^***2***^	***df***	***χ*** ^***2***^ ***/df***	***∆χ*** ^***2***^ ***(∆df)***	***CFI***	***TLI***	***IFI***	***RMSEA***
Five-factor model	292.54	142	2.06	Baseline model	0.97	0.96	0.97	0.06
Four-factor model	882.44	146	6.04	589.90^***^(4)	0.85	0.82	0.85	0.13
Three-factor model	1785.37	149	11.98	1492.83^***^(7)	0.66	0.61	0.66	0.19
Two-factor model	2342.37	151	15.51	2049.83^***^(9)	0.55	0.49	0.55	0.22
One-factor model	3359.96	152	22.11	3067.42^***^(10)	0.34	0.25	0.34	0.26
Zero model	4997.89	171	29.23					

Five-factor model: leader humor, employee creativity, creative self-efficacy, traditionality, positive affect; Four-factor model: leader humor + employee creativity, creative self-efficacy, traditionality, positive affect; Three-factor model: leader humor + employee creativity, creative self-efficacy + traditionality, positive affect; Two-factor model: leader humor + employee creativity, creative self-efficacy + traditionality + positive affect; One-Factor Model: Two-factor model: leader humor + employee creativity + creative self-efficacy + traditionality + positive affect. “+” combing the factors.

*** *p* < 0.001.

The correlation coefficients, means, and standard deviations of leader humor, creative self-efficacy, traditionality, and employee creativity are presented in Table 3. Creativity self-efficacy (*r* = 0.55, *p < 0*.01) and positive emotion (*r* = 0.20, *p* < 0.01) has a significant positive relation to employee creativity. Gender (*r* = 0.01, *p* > 0.05), age (*r* = −0.05, *p* > 0.05), and education level (*r* = 0.11, *p* > 0.05) has no significant correlation with employee creativity. Job tenure has a significant positive relation to employee creativity (*r* = 0.14, *p* < 0.05) but has no significant correlation with employee creative self-efficacy (*r* = 0.10, *p* > 0.05).

**Table 3:** Results of descriptive statistical analysis and coefficients of correlation.

**Variables**	**Mean**	**SD**	**1**	**2**	**3**	**4**	**5**	**6**	**7**
***Level 1***									
1. Gender	0.49	0.50							
2. Age	2.27	1.04	−0.05						
3. Education level	2.69	0.83	0.16^**^	−0.05					
4. Job tenure	2.41	0.89	−0.07	0.18^**^	0.12^*^				
5. Positive affect	3.07	0.72	0.01	0.01	0.07	0.14^**^			
6. Creative self-efficacy	3.98	0.83	−0.08	0.04	0.04	0.10	0.15^**^		
7. Traditionality	3.04	1.17	0.04	−0.02	−0.03	−0.12^*^	−0.001	0.22^**^	
8. Employee creativity	4.13	0.70	0.01	−0.05	0.11	0.14^*^	0.20^**^	0.55^**^	0.11
***Level 2***									
1. Leader humor	4.66	0.70							

N (Level 2) = 88; N (Level 1) = 306.

* *p* < 0.05;

** *p* < 0.01.

### The main and mediating effects test

There were significant between-group differences in creativity self-efficacy (χ^2^_(87)_ = 212.21, *p* < 0.001) and employee creativity (χ^2^_(87)_ = 226.29, *p* < 0.001). Therefore, a multi-level hierarchical linear model (HLM) was used to analyze the data (please see results in Table 4). To test the main effect, gender, age, education level, job tenure, positive emotions, and leader humor were simultaneously entered into the regression equation with employee creativity as the dependent variable. Model 2 showed that the correlation coefficient between leader humor and employee creativity is 0.38 at the 0.001 significance level (*β* = 0.38, *p* < 0.001). So, H1 was supported. Next, to test the mediating effect, gender, age, education level, job tenure, positive emotions, leader humor, and creative self-efficacy were simultaneously entered into the regression equation with employee creativity as the dependent variable. Model 3 showed the positive effect of creative self-efficacy on employee creativity (*β* = 0.43, *p* < 0.001), while the positive effect of leader humor on employee creativity is no longer significant (*β* = 0.15, *p* > 0.05). So, it can be inferred that creative self-efficacy is a significant mediating variable in the link between leader humor and employee creativity, thus H2 was supported.

**Table 4:** Results of hierarchical linear modeling.

	**Employee creativity**			**Creative self-efficacy**	
	*Model 1*	*Model 2*	*Model 3*	*Model 4*	*Model 5*
Intercept	4.17^***^(0.04)	4.14^***^(0.04)	4.13^***^(0.04)	3.97^***^(0.04)	4.00^***^(0.04)
***Level 1***					
Gender	0.01(0.07)	0.03(0.06)	0.09(0.05)	−0.10(0.09)	−0.12(0.08)
Age	−0.05(0.04)	−0.05(0.03)	−0.04(0.03)	0.01(0.03)	0.01(0.03)
Education level	0.03(0.04)	0.03(0.04)	0.04(0.04)	−0.01(0.05)	−0.02(0.04)
Work tenure	0.08(0.04)	0.09(0.05)	0.05(0.04)	0.09(0.05)	0.09^*^(0.05)
Positive emotion	0.12(0.06)	0.09(0.06)	0.05(0.05)	0.10(0.08)	0.07(0.07)
Creative self-efficacy			0.43^***^(0.05)		
Traditionality					0.07^*^(0.04)
***Level 2***					
Leader humor		0.38^***^(0.09)	0.15(0.08)	0.60^***^(0.08)	0.48^***^(0.09)
Interaction					
Leader humor × Traditionality					−0.16^*^(0.06)
*ΔR* ^2^	0.25	0.33	0.50	0.40	0.46

N (Level 2) = 88; N (Level 1) = 306. Unstandardized coefficients are presented, with the corresponding standard errors in parentheses.

* *p* < 0.05;

*** *p* < 0.001.

Then, bootstrap method was applied to further test the role of creative self-efficacy in mediating the process by which leader humor influences employee creativity. The results show that the indirect effect of leader humor on employee creativity through creative self-efficacy was 0.26, with a 95% confidence interval (*CI*) of [0.17, 0.35], excluding 0. The mediator role of creative self-efficacy is significant once again. Thus, H2 was fully supported.

### The moderating effects test

**Figure 2:**
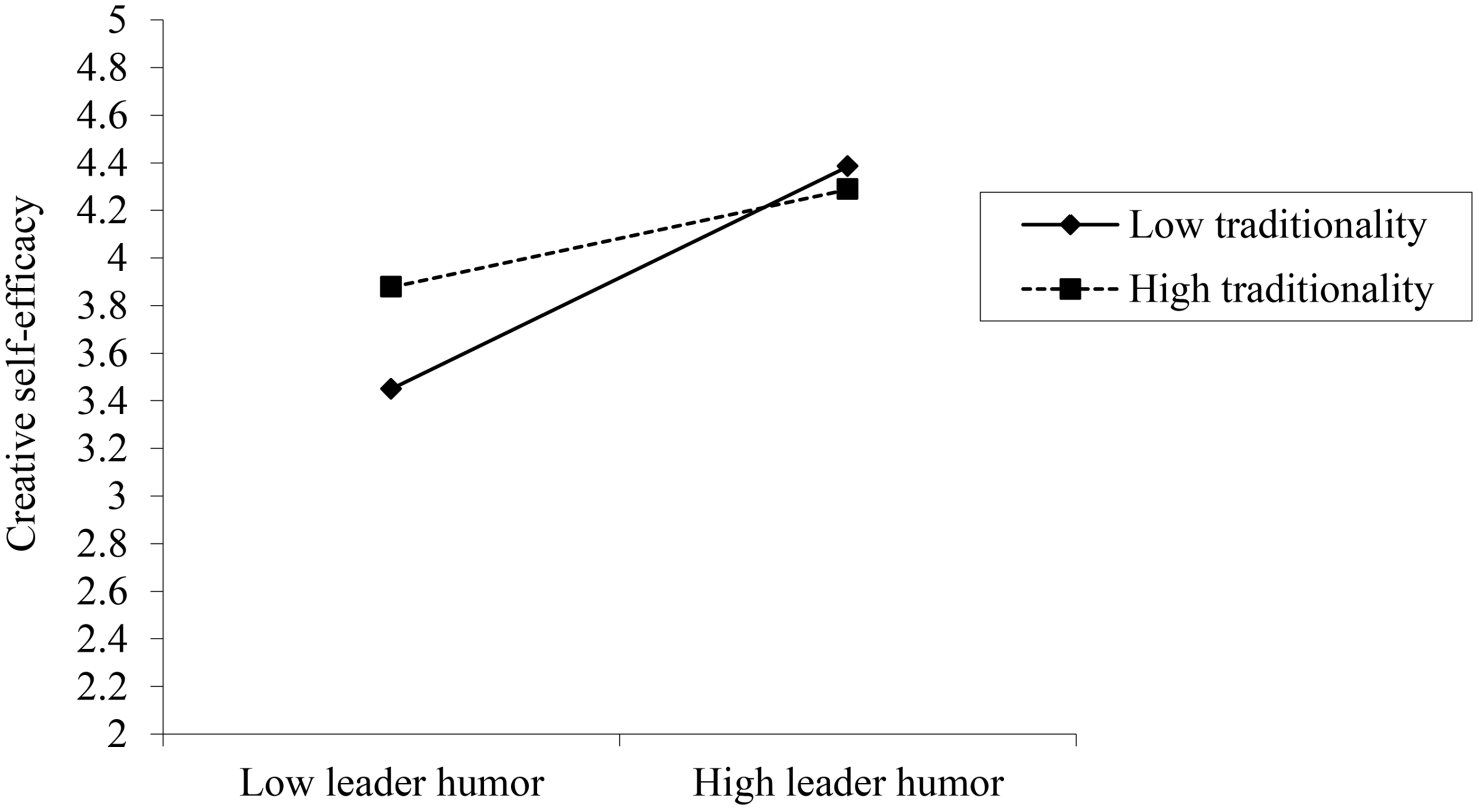
The moderating effect of traditionality.

To test the moderating effect, gender, age, education level, job tenure, positive emotions, leader humor, traditionality, and interaction terms were simultaneously entered into the regression equation with creative self-efficacy as the dependent variable. According to Model 5 (as shown in Table 4), the interaction term of leader humor and traditionality has a significant negative impact on creative self-efficacy (*β* = −0.16, *p* < 0.001), proving that traditionality negatively moderates the connection between leader humor and creative self-efficacy. Then, a simple slope test was also performed (please see the moderating effect plot of traditionality in Figure 2). The results showed that the positive impact of leader humor on creative self-efficacy was stronger when employee traditionality was low (*β* = 0.67, *p* < 0.001) than employee traditionality was high (*β* = 0.29, *p* < 0.05), offering initial support for H3.

Next, bootstrap method was conducted to test the moderated mediation effect. Table 5 showed that the positive association between leader humor and creative self-efficacy was significant both at high (*β* = 0.29, 95% *CI* = [0.07, 0.52]) and low (*β* = 0.67, 95% *CI* = [0.44, 0.89]) levels of traditionality. There are significant differences between high and low traditionality levels (*β* = −0.38, 95% *CI* = [−0.65, −0.10]). Thus, H3 was fully supported. Then, it can also be seen in Table 5 that the indirect effect of creative self-efficacy on the association between leader humor and employee creativity was significant either at high (*β* = 0.12, 95% *CI* = [0.03, 0.22]) or low (*β* = 0.27, 95% *CI* = [0.17, 0.39]) traditionality level, the difference (*β* = −0.15, 95% *CI* = [−0.28, −0.064]) between high and low level of traditionality was also significant, which suggests that the mediating role of creative self-efficacy in the process of leader humor impacts employee creativity is moderated by individuals traditionality, thus, supporting H4.

**Table 5:** Results of moderated mediation analysis.

**Moderate variable**	**Leader humor→Creative self-efficacy**	**Indirect effect**	**Direct effect**	**Total effect**
High traditionality	0.29^*^ [0.07, 0.52]	0.12^*^ [0.03, 0.22]	−0.04 [−0.22, 0.14]	0.08 [−0.13, 0.28]
Low traditionality	0.67^*^ [0.44, 0.89]	0.27^*^ [0.17, 0.39]	0.28^*^ [0.11, 0.46]	0.55^*^ [0.35, 0.77]
Differences(*Δ*)	−0.38^*^ [−0.65, −0.10]	−0.15^*^ [−0.28, −0.04]	−0.32^*^ [−0.56, −0.10]	−0.47^*^ [−0.74, −0.22]

N (Level 2) = 88; N (Level 1) = 306; 95% confidence interval in parentheses.*p < 0.05.

## Discussion

The present study examined the impact of leader humor on employee creativity and contributes to the development of research on the relationship between leader humor and employee creativity. First, although, there have been previous studies on the relationship between leader humor and employee creativity, but they put leaders and employees at the same level for research. Leaders and employees belong to different levels of the organization. Thus, making a good case for cross-level analysis to better explain the relationship between them. The multi-level examination of leader humor and employee creativity was conducted to test the study hypotheses. The results showed that the leader humor can positively affect employee creativity. Second, the individual’s self-concept has an important influence on employee behavior, but the current mediating mechanism of leader humor on employees’ creativity focused on expounding the role of individual’s emotions (Cooper et al., 2018), and psychological capital (Li et al., 2019), ignoring the important influence of individual self-concept. As an important individual concept, self-efficacy may be overlooked as the mediating role between leader humor and employee creativity. Therefore, we examined the mediating role of creative self-efficacy which demonstrated that creative self-efficacy exerts a significant mediating effect in the process of leader humor in fostering employee creativity. Finally, previous scholars have examined the relationship between leader humor and employee creativity from the perspective of social exchange mainly. Therefore, the selection of contextual variables is considered from the perspective of social exchange, such as perspective-taking (Hu and Luo, 2020), and employees’ sensitivity to favorable interpersonal treatment (Peng et al., 2020). Drawing upon cultural self-representation theory we argue that traditional values have an important impact on the relationship between leader humor and employee creativity. The results showed that employee traditionality plays a significant moderating role in the relationship between leader humor and employee creativity, and also moderates the indirect effect of leader humor on employee creativity through creative self-efficacy.

### Theoretical implications

Our study presents the following theoretical directions. First, the present study broadens the underpinning theory, its application and scope while also deepening our understanding of the socio-psychological mechanism of leader humor affecting employees’ creativity. Existing scholars usually define leader humor from the perspective of social exchange and explain the relationship between leader humor and creativity (Lang and Lee, 2010; Cooper et al., 2018). However, they believe that leader humor can adjust employees’ cognition by providing them with positive emotional and psychological experiences, reduce their stress, improve their interpersonal skills, and thus, promoting employees’ creativity. As a result, humor research based on social exchange theory believes that the focus of leader humor is interpersonal communication. On the contrary to this, leader behavior, as one of the main work environments that employees come into contact with, has more than just interpersonal effects on employees. Based on the cultural self-representation theory, we tested the mechanism by which leader humor affects employee creativity, and it is hypothesized that employee creativity is influenced by the interaction of people and the environment. As an important work environment, leader humor has an effect on creativity by affecting employees’ perception of individual self-efficacy and is constrained by individual traditional values. Hence, this study broadens the range of applicable scenarios for leader humor, deepens the understanding of the relationship between leader humor and employee creativity, and promotes the development of the socio-psychological mechanism by which leader humor influences employee creativity.

Second, this study encourages not only the research on the cross-level mechanism of leader humor on employee creativity but also research on the occurrence mechanism of employee creativity. Although the role of leadership style in fostering employee creativity has received considerable attention on the transformational leadership (Shafi et al., 2020), innovation leadership (Kremer et al., 2019), empowering leadership (Zhang and Zhou, 2014), moral leadership (Gu et al., 2015), servant leadership (Jaiswal and Dhar, 2017), and authentic leadership (Guo et al., 2018), little research has explored the connection between humorous leadership and employee creativity. The present study examined the cross-level effects of leader humor on employee creativity from the perspective of cultural self-representation theory. The findings show that leader humor has an impact on creativity through individual creative self-efficacy, which verifies the theoretical hypothesis of cultural self-representation theory that the environment and people work together to promote creativity. Therefore, this study contributes to a better understanding of the relationship between leader humor and employee creativity while also encouraging the development of mechanism that promote employee creativity.

Third, drawing upon the cultural self-representation theory, we introduced the mediating role of creative self-efficacy in the process by which leader humor influences employee creativity and promotes the research on the mediating mechanism of leader humor affecting employee creativity. Recently, Hu and Luo (2020) constructed an integrated model to explore the effect of workplace humor on employee creativity and found that task resources and affective commitment act as an important mediating role in the process. Peng et al. (2020) suggested that the role of employees themselves is an important mediating mechanism for leader humor to affect creativity. Besides, relational energy (Yang et al., 2021) and psychological capital (Li et al., 2019) also affects the process of leader humor affecting employee creativity. However, these studies ignore the impact of employees’ creative traits on creativity. This study interprets the relationship between leader humor and employee creativity from the perspective of cultural self-representation theory. Cultural representation theory emphasizes that the work environment can influence individual behavior by influencing an individual’s self-concept. Creativity self-efficacy, as a self-concept, is further affected by leader behavior, and as an individual creative trait, it will affect creativity. Therefore, this study focused on the mediating role of creative self-efficacy between leader humor and employee creativity. The empirical results showed that the creative self-efficacy partially mediates the process of leader humor influencing employee creativity, which promotes the research on the mediating mechanism by which leader humor affects employee creativity, and expands the application scenarios and scope of cultural self-representation theory.

Finally, according to the role of cultural values emphasized in the cultural self-representation theory, traditionality is introduced as a situational factor to moderate the psychological mechanism of leader humor in promoting employee creativity through creative self-efficacy. Therefore, the boundary condition research on leader humor influences employee creativity is promoted. Previous research shows that employees’ sensitivity to favorable interpersonal treatment (Peng et al., 2020), perspective taking (Hu and Luo, 2020), trust (Lee, 2015), supervisor-subordinate dyadic tenure and work autonomy (Li et al., 2019) can moderate the mediating mechanism of leader humor’s impact on employee creativity. However, existing studies rarely noticed the important role of cultural values in the process of leader humor impacting employee creativity *via* creative self-efficacy. The empirical results show that employee traditionality not only negatively moderates the positive relationship between leader humor and employee creative self-efficacy, but also moderates the indirect influence of creative self-efficacy in the link between leader humor and employee creativity. This study has thus, contributed to the development of the humor and creativity literature by advancing the boundary condition research on the influence of leader humor on creative self-efficacy and creativity.

### Practical implications

The findings of this study have the following practical implications for managers. Firstly, managers should be more aware of the impact that humorous behavior has on employee creativity. This study found that leaders’ humorous behaviors can directly or indirectly (*via* creative efficacy) promote employee creativity, indicating that leader humor is instrumental in enhancing employee creativity. Therefore, organizations can properly publicize the significance of leaders’ humorous behaviors, and implement some practical measures to promote humor in leadership. For existing leaders, (1) organizations can provide training to improve their ability to communicate with employees through humorous language or behavior, such as hiring humorous mentors to demonstrate and guide on-site or organizing leaders to watch the video of the daily behavior of people with humorous characteristics; (2) leaders can also develop their own sense of humor through “smiles,” such as smiling in the mirror every day, reading more interesting books, listening to interesting stories, and approaching interesting people to cultivate their own sense of humor; (3) at the same time, it is necessary to cultivate close relationships with subordinates and break the feudal ideology of “official standard,” so as to improve employees’ acceptance of leadership humor. For the selection of leaders, those employees who are outgoing and humorous can be cultivated as key candidates for promotion. And specifically, humor tests and personality tests can be conducted during the recruitment and selection process of managers to observe their humor potential.

Secondly, the results showed that creative self-efficacy act as a significant mediator between leader humor and employee creativity, suggesting that creative self-efficacy is a key factor in stimulating employee creativity. Therefore, organizations can take measures to promote employees’ creative self-efficacy. From the perspective of work-related factors, task autonomy, perceived levels of support for creativity (Mathisen, 2011), and creativity training (Vally et al., 2019) can all improve employees’ creative self-efficacy. Therefore, organizations can increase the autonomy of employees’ work tasks, raise the level of support for employees’ creative results, and organize creativity training on employees to foster their creative self-efficacy; From the perspective of work relationship-related factors, knowledge sharing (Hu and Luo, 2020), peer review (Liu et al., 2016), and high-quality leader-employee relationships (Mathisen, 2011) also play an important role to promote employees’ creative self-efficacy. Therefore, organizations can maintain a good relationship between leaders and employees and among colleagues, and promote knowledge sharing by providing a relaxed and tolerant atmosphere to foster high-quality work relationships in organizations. Besides, leaders should encourage employees in a timely manner. The timely encouragement of leaders to employees can greatly promote employees’ fighting spirit and improve self-efficacy. When employees experience failure, leaders should give encouragement and affirmation in a timely manner to stimulate employees’ inner potential. When employees experience success, leaders can inspire people in a timely manner, pursue the victory, let employees truly experience the feeling of success, and turn this feeling into inner strength and belief.

Thirdly, this study proves that traditionality negatively moderates the impact of leader humor on creative self-efficacy, and that creative self-efficacy plays a mediating role in the process of leader humor impacting employee creativity, suggesting that traditionality hinders the promotion of employee creative self-efficacy and creativity. For employees who are deeply influenced by traditional values, to enhance employee creativity, leaders can differentiate between highly traditional employees and lowering their traditional attitudes by providing career counseling and values-based clarification training (Guan et al., 2016). At the same time, it is necessary to promote employees who combine cultural values with their work and life goals, integrate cultural values with the needs of the development of the new era, and enhance their creative adaptability at work. Additionally, leaders can encourage employees to make changes. By citing some examples of companies and employees being eliminated by society and the market due to conformity, leaders can promote employees to realize the importance of change and innovation. For example, employees in traditional manual workshops are being replaced by robots and artificial intelligence. If changes are not made on time, they will be eliminated by society. For employees with a low level of traditionality, leaders can use humorous language and behavior to communicate with them appropriately to cultivate high-quality leader-employee relationships and promote employees’ creative self-efficacy and creativity.

### Limitation and future recommendations of research

Our study has the following limitations: To begin with, the samples in this study come from companies in the automotive, IT, and pharmaceutical industries, all of which place a high value on employee creativity. As a result, the findings of this study are applicable to industries and enterprises with high creative requirements, and research should exercise caution when applying the findings of this study to firms in other industries. Future research can broaden the study sample to improve the generalizability of the findings. Next, this study only looks at the role of creative and self-efficacy in mediating the relationship between leader humor and employee creativity. Future research should look at the role of functions-relationship building and stress-related constructs like stress and workplace anxiety. Finally, the sample of this study is limited to the data of employees from Chinese firms, so, it is not clear how much of the results can be generalized to Western context. Because traditionality has been linked with Chinese culture, it will be more important for future studies to further verify the moderating role of traditionality from varied cultures. Meanwhile, traditionality is only one of many traditional cultural concepts of a country or society, and future research can focus on other traditional cultural values’ roles in the link of leader humor with employee creativity. For example, cultural factors, such as power distance, collectivism may also influence followers’ receptiveness to leader humor.

## Conclusion

Leader humor substantially helps in fostering employee creativity. Drawing upon the cultural representation theory, this study examined the role of leader humor in promoting employee creativity by introducing two key factors, creative self-efficacy, and traditionality. The findings showed that traditionality is a significant moderator in the relationship between leader humor and employee creativity through the lens of creative self-efficacy. This study offers fresh insights into how leader humor affects creativity, promotes creativity research, and informs managers about the importance of fostering employee creativity.

## Data availability statement

The original contributions presented in the study are included in the article/supplementary material; further inquiries can be directed to the corresponding author.

## Ethics statement

Ethical approval was not provided for this study on human participants because an ethics approval was not required as per our institution’s guidelines and national regulations. The patients/participants provided their written informed consent to participate in this study.

## Author contributions

YZ: conceptualization and writing-original draft. CY: data curation, writing-review, and editing. MA: data analysis, writing-review, and editing. YW: writing-review and editing. All authors contributed to the article and approved the submitted version.

## Funding

We acknowledge the financial support from the Humanities and Social Sciences of Ministry of Education Planning Fund in China (18YJA630149).

## Conflict of interest

The authors declare that the research was conducted in the absence of any commercial or financial relationships that could be construed as a potential conflict of interest.

## Publisher’s note

All claims expressed in this article are solely those of the authors and do not necessarily represent those of their affiliated organizations, or those of the publisher, the editors and the reviewers. Any product that may be evaluated in this article, or claim that may be made by its manufacturer, is not guaranteed or endorsed by the publisher.
